# Postnatal growth retardation is associated with intestinal mucosa mitochondrial dysfunction and aberrant energy status in piglets

**DOI:** 10.1111/jcmm.15621

**Published:** 2020-07-15

**Authors:** Ming Qi, Jing Wang, Bie Tan, Simeng Liao, Cimin Long, Yulong Yin

**Affiliations:** ^1^ Laboratory of Animal Nutritional Physiology and Metabolic Process Key Laboratory of Agro‐ecological Processes in Subtropical Region National Engineering Laboratory for Pollution Control and Waste Utilization in Livestock and Poultry Production Institute of Subtropical Agriculture Chinese Academy of Sciences Changsha China; ^2^ University of Chinese Academy of Sciences Beijing China; ^3^ Hunan International Joint laboratory of Animal Intestinal Ecology and Health Laboratory of Animal Nutrition and Human Health College of Life Sciences Hunan Normal University Changsha China; ^4^ College of Animal Science and Technology Hunan Agricultural University Changsha China

**Keywords:** energy metabolism, intestinal mucosa, mitochondrial dysfunction, nutrient absorption, pig, postnatal growth retardation

## Abstract

Individuals with postnatal growth retardation (PGR) are prone to developing chronic disease. Abnormal development in small intestine is casually implicated in impaired growth performance. However, the exact mechanism is still unknown. In this present study, PGR piglets (aged 42 days) were employed as a good model to analyse changes in nutrient absorption and energy metabolism in the intestinal mucosa. The results showed lower serum concentrations of free amino acids, and lipid metabolites in PGR piglets, which were in accordance with the down‐regulated mRNA expressions involved in fatty acid and amino acid transporters in the jejunal and ileal mucosa. The decreased activities of digestive enzymes and the marked swelling in mitochondria were also observed in the PGR piglets. In addition, it was found that lower ATP production, higher AMP/ATP ratio, deteriorated mitochondrial complex III and ATP synthase, and decreased manganese superoxide dismutase activity in the intestinal mucosa of PGR piglets. Furthermore, altered gene expression involved in energy metabolism, accompanied by decreases in the protein abundance of SIRT1, PGC‐1α and PPARγ, as well as phosphorylations of AMPKα, mTOR, P70S6K and 4E‐BP1 were observed in intestinal mucosa of PGR piglets. In conclusion, decreased capability of nutrient absorption, mitochondrial dysfunction, and aberrant energy status in the jejunal and ileal mucosa may contribute to PGR piglets.

## 1 INTRODUCTION

Postnatal growth retardation (PGR) is associated with lifelong consequences beyond reduced weight, including metabolic disturbance and impaired immune function.^1^ Postnatal growth retardation piglets had impaired hormone profiles, antioxidant system and inflammatory responses.^2^, ^3^ The small intestine plays an important role in terminal digestion and absorption of nutrients during postnatal growth in animals.^4^ In response to various stress (eg pathogen, change in nutrition, new environment), the small intestine undergoes marked changes, including accelerated tissue growth and functional maturation during the immediate postnatal period.^5^, ^6^ The marked changes of gut function are thought to be critical for the poor performance observed.^7^ The deteriorated intestinal mucosal barrier function has been observed in PGR piglets as compared with healthy piglets.^8^, ^9^ Of note, abnormal digestion and nutrition absorption is one of the critical factors causing growth retardation.^10^

Previously, our transcriptome analysis of intestinal samples from healthy and PGR piglets revealed differential expression of genes that were involved in metabolic pathway.^8^ The present study was conducted to further investigate the specific characteristics of energy metabolism associated with PGR. A large number of studies have shown anatomical changes in the intestine, such as reduced villus height, lower enzyme activity, as well as smaller area of passive absorption in the piglets with intrauterine growth retardation (IUGR).^11^, ^12^, ^13^ Impaired intestinal function of absorption, leading to stunt, exacerbate pre‐existing deficiencies through a reduction in food intake and increased nutrient needs.^14^ Inadequate intakes of energy, protein, or micronutrients cause disorder of energy metabolism and may result in growth retardation.^15^, ^16^ In addition, recent studies have been directed to mitochondrial dysfunction as a potential factor. It is well known that mitochondria are the major sites of energy metabolism and reactive oxygen species production.^17^ The intestinal antioxidant enzymes and mRNA abundances involved in mitochondrial function such as mitochondrial transcription factor A (TFAM) were affected by PGR.^18^ Intrauterine growth retardation impaired the mitochondrial biogenesis and activities of electron transfer chain (ETC) complexes in human and animal models, which may be associated with disturbed signalling pathway involved in energy metabolism, such as AMP‐activated protein kinase (AMPK) pathway.^19^, ^20^, ^21^ Previous liver proteome analysis from healthy and IUGR piglets also found majority of differently expressed proteins that were involved in mitochondrial function and glucose and lipid metabolism.^22^ Thus, early nutritional intervention to improve mitochondrial function and energy metabolism may be an effective treatment for PGR piglets.

Therefore, digestive enzyme activities, nutrient transporters expressions, mitochondrial function and signalling pathway involved in energy metabolism in the jejunal and ileal mucosa of PGR piglets were investigated in the present study. Results from the present study may also help to establish preventive strategies for disorder of intestinal mucosal energy metabolism of the PGR piglets and individuals.

## 2 MATERIALS AND METHODS

### 2.1 Animals and experimental design

All animals used in this study were humanely managed according to the Chinese Guidelines for Animal Welfare. The experimental protocol was approved by the Animal Care and Use Committee of the Chinese Academy of Sciences (Beijing, China) (IACUC # 201 302).

A group of Duroc × Landrace × Large Yorkshire crossbred male pigs with the same paternal origin and maternal health condition were used in this study. These pigs weaned at 21 days of age were housed in the same environment and fed the same commercial feeds.^2^ At 42 day of age, six PGR pigs (body weight, BW 5.40 ± 0.38 kg) and six healthy pigs (BW 11.01 ± 0.40 kg) that were pair‐matched by litter were selected for sampling. PGR pigs were defined as the pigs with BW lighter than 70% of average litter BW and had no obvious characteristics of disease or injury according to the previous study.^2^ Five mL of blood was collected in aseptic capped tubes from a jugular vein in the morning after overnight fasting, centrifuged at 2000 × *g* for 10 minutes at 4°C to obtain serum samples,^23^ and stored at −80°C for further analysis. After electrical stunning, piglets were killed and the small intestine was rinsed thoroughly with ice‐cold 0.9% sterile saline solution. The middle segments of the jejunum (2 cm), and ileum (2 cm) were cut and fixed in 2.5% glutaraldehyde for transmission electron microscopy (TEM) studies. A fraction of fresh intestinal mucosa sample was scraped and then rapidly treated for the isolation of mitochondria using tissue mitochondria isolation kit (Beyotime Institute of Biotechnology) according to the manufacturer's instructions. The remaining parts were immediately snap‐frozen in liquid nitrogen and stored at −80°C for determining of enzyme activity, RNA extraction and Western blot analysis.

### 2.2 Determination of blood biochemical parameters

Serum biochemical parameters, including triglyceride (TG), total cholesterol (CHOL), high‐density lipoprotein (HDL), low‐density lipoprotein (LDL), calcium and phosphorus were measured using an instrument (Biochemical Analytical Instrument, Beckman CX4, Beckman Coulter Inc) and commercial kits (Sino‐German Beijing Leadman Biotech Ltd.) in accordance with the manufacturer's instructions.

### 2.3 Determination of serum free amino acids

The 8.0% sulphosalicylic acid dihydrate solution (600 μL) was added to 600 μL serum and mixed thoroughly. This mixture was centrifuged at 10 000 *g* and 4°C for 10 minutes. The supernatant was collected and applied to an ion‐exchange amino acid analyzer (Hitachi L‐8900 Auto‐Analyzer) for the determination of amino acids.

### 2.4 Transmission electron microscopy analysis

Small sections of the jejunum and ileum were used for TEM studies.^24^ Tissue segments were fixed in 3.0% buffered glutaraldehyde, washed with phosphate‐buffered saline (PBS) and then post‐fixed in 1% osmium tetroxide for 2 hours at 4°C. Then segments were dehydrated with an increasing gradient of ethanol for 10 minutes at room temperature and embedded in EPOK 812 (Oukenn). Semi‐thin (0.5 µm) and ultra‐thin (90 nm) sections were cut, mounted on copper grids, and post‐stained with uranyl acetate and lead citrate at room temperature for 20 minutes. The stained thin sections were then examined using a TEM (H‐600IV; Hitachi High‐Technologies Corp.), and images were analysed using IPP 6.0 software (International Business Machines Corporation, IBM) following previous study.^25^

### 2.5 Measurement of mitochondrial enzyme activity

Activities of the ETC complexes I, II, III, IV and adenosine triphosphate (ATP) synthase in the jejunal and ileal mucosa were measured using commercial kits (Jiangsu Yutong Biological Technology Co., Ltd.). The protein content of the intestinal mucosa was measured using the bicinchoninic acid (BCA) assay method (Beyotime Institute of Biotechnology). The mitochondrial enzyme activity was normalized to the protein concentration (U/g) of whole‐cell lysates (WCLs).

### 2.6 Measurement of intestinal mucosal energy status

The adenosine monophosphate (AMP), adenosine diphosphate (ADP) and ATP production in jejunal and ileal mucosa from healthy and PGR piglets were measured using commercially available swine AMP, ADP enzyme‐linked immunosorbent assay (ELISA) kits (Jiangsu Yutong Biological Technology Co., Ltd.) and ATP ELISA kit (Nanjing Jiancheng Bioengineering Institute), respectively. Adenosine levels were normalized to the protein concentration (nmoL/g) of WCLs. Finally, the AMP/ATP ratio was calculated, and the energy charge (EC) value was calculated using the following equation: EC = (ATP + 0.5 ADP)/ (ATP + ADP + AMP).

### 2.7 Determination of mitochondrial redox status

The fresh intestinal mucosal mitochondria were isolated using tissue mitochondria isolation kit (Beyotime Institute of Biotechnology) according to the manufacturer's instructions. The activities of manganese superoxide dismutase (Mn‐SOD), glutathione peroxidase (GPx) and the concentrations of malondialdehyde (MDA) were determined using commercial kits (Nanjing Jiancheng Bioengineering Institute).

### 2.8 Analysis of intestinal mucosal digestive enzyme activities

Mucosal tissue samples from the jejunum and ileum were homogenized in ice‐cold PBS and centrifuged at 6000 × g for 20 minutes at 4°C, and the supernatant was used for enzyme analysis. The activities of intestinal alkaline phosphatase (ALP), lactase, sucrase, and maltase were analysed using commercially available swine ELISA kits according to the manufacturer's instructions (Jiangsu Yutong Biological Technology Co., Ltd.). The protein contents of WCLs were measured using the BCA assay method (Beyotime Institute of Biotechnology). The intestinal digestive enzyme activity was normalized to the protein concentration (mU/mg) of WCLs.

### 2.9 Real‐time quantitative PCR

Total RNA was isolated from the liquid nitrogen‐pulverized intestinal mucosa samples with the TRIZOL reagent (Invitrogen) following the manufacturer's protocol and quantified by electrophoresis on 1% agarose gel. DNA‐free RNA (1 μg) was used for reverse transcription. cDNA was synthesized with 5 × PrimeScript Buffer2 and PrimeScript reverse transcriptase Enzyme Mix 1 (Takara Biotechnology Co., Ltd). Primers (Table S1) were designed with Primer 5.0 (PREMIER Biosoft International) according to the gene sequence of the pig to produce an amplification product, as described previously.^26^ The primers used to amplify genes are shown in Table S1. The real‐time quantitative PCR was performed in a volume of 10 μL (LightCycler^®^ 480 Real‐Time PCR System, Roche) as described previously.^8^ The relative expression levels of the selected genes normalized against the reference gene (*β‐actin*) were calculated by using the 2^－△△Ct^ method.^27^ Data are expressed as the relative values to those for healthy pigs.

### 2.10 Western blot analysis

Jejunal and ileal mucosa samples were homogenized with radio immunoprecipitation assay (RIPA) lysis buffer (Beyotime Institute of Biotechnology), and the protein concentrations were determined using a BCA assay kit (Beyotime Institute of Biotechnology). The Western blot procedure was according to the method described previously.^28^ The relative protein levels for AMPKα, phosphor (P)‐AMPKα, silent information regulator transcript 1 (SIRT1), peroxisome proliferators‐activated receptor γ (PPARγ), peroxisome proliferator‐activated receptor gamma coactivator‐1α (PGC‐1α), mammalian target of rapamycin (mTOR), P‐mTOR, P70 S6 kinase (P70S6K), P‐P70S6K, eukaryotic initiation factor 4E‐binding protein 1 (4E‐BP1), P‐4E‐BP1 were determined by Western blot technique as described previously.^29^ The following antibodies were used for protein quantification: anti‐AMPKα (1:1000; Cell Signaling Technology, MA, US); anti‐P‐AMPKα (1:1000; Cell Signaling Technology); anti‐SIRT1 (1:1000; Bioss, Inc, Woburn, MA, US); anti‐PPARγ (1:1000; Bioss, Inc); anti‐PGC‐1α (1:1500; Proteintech, IL, US); anti‐mTOR (1:500; Cell Signaling Technology); anti‐P‐mTOR (1:1000; Abcam, Cambridge, UK); anti‐P70S6K (1:1000; Abcam); anti‐P‐P70S6K (1:1000; Cell Signaling Technology); anti‐4E‐BP1 (1:1000; Cell Signaling Technology); anti‐P‐4E‐BP1 (1:1000; Cell Signaling Technology); anti‐β‐actin (1:5000; Proteintech) along with secondary antibody horseradish peroxidase‐conjugated goat anti‐rabbit or mouse IgG (1:5000; ZSGB Biological Technology, Beijing, China). All protein measurements were normalized to β‐actin, and data were expressed relative to the values in healthy piglets.

### 2.11 Statistical analysis

All statistical analysis was performed by the independent sample t test using SPSS software 23.0 (SPSS Inc). Statistical model included treatment as fixed effect and animal as experimental unit. Data were expressed as means ± SEM *P*‐values <.05 were taken to indicate statistical significance. Cohen's *d* and post hoc testing for *t* test were used to determine the effect size and the statistical power, respectively.

## 3 RESULTS

### 3.1 Decreased nutrients absorption in PGR piglets

**FIGURE 1:**
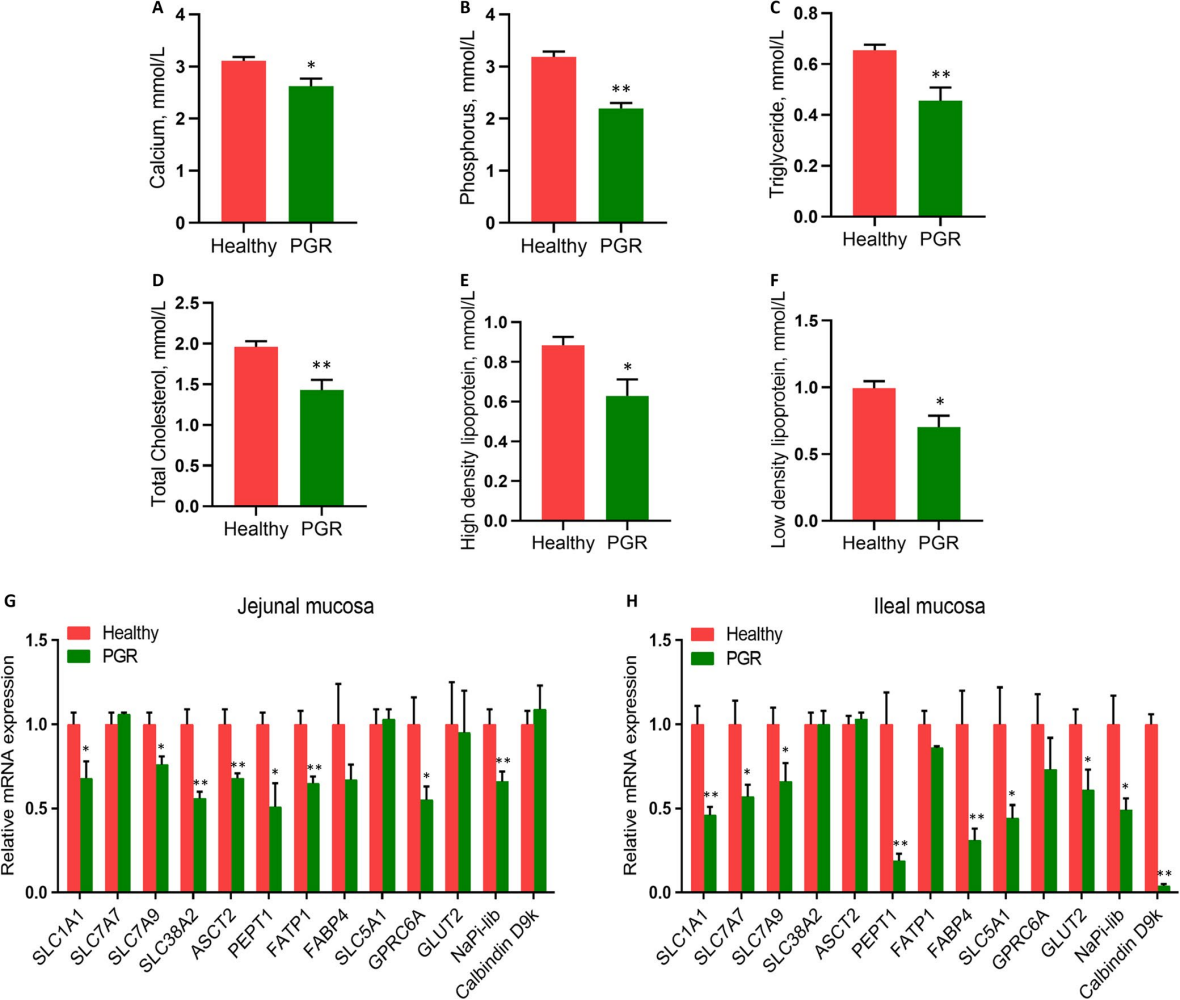
Changed nutrient absorption in healthy and PGR piglets. A‐F, serum biochemical parameters; (G, H) mRNA expression of nutrient transporters in jejunal (G) and ileal mucosa (H). Data are expressed as means ± SEM, n = 6. *, *P* < .05; **, *P* < .01

We determined the ability of nutrient absorption in PGR piglets. As shown in Figure 1, significantly decreased serum concentrations of TG, CHOL, HDL, LDL, calcium and phosphorus were observed in PGR piglets as compared to healthy piglets (*P* < .05). Additionally, 10 of 18 serum free amino acids we monitored showed significant differences between PGR and healthy piglets (*P* < .05). The serum concentrations of glycine (Gly), tyrosine (Tyr), arginine (Arg), proline (Pro), aspartate (Asp), threonine (Thr), serine (Ser), alanine (Ala), cysteine (Cys) and leucine (Leu) in PGR piglets were significantly decreased compared with the healthy piglets (*P* < .05). There was no significant difference in the concentrations of glutamate (Glu), valine (Val), methionine (Met), isoleucine (Ile), phenylalanine (Phe), lysine (Lys), histidine (His) and Tryptophan (Trp) (*P* > .05) (Table 1). In accordance with the decreased serum concentration of nutrients, the mRNA expression involved in nutrient transporters in intestinal mucosa showed same trend in PGR piglets. Several amino acid transporters (SLC1A1, SLC7A7, SLC7A9, SLC38A2 and ASCT2), peptide transporters (PEPT1), fatty acids transporters (FATP1 and FABP4), glucose transporters (SLC5A1, GPRC6A and GLUT2) and mineral transporters (NaPi‐lib, Calbindin D9k) in the jejunal and ileal mucosa were tested (Figure 1). In the jejunal mucosa, marked down‐regulation of mRNA expression of all tested amino acids transporters except for *SLC7A7*, *FATP1*, *GPRC6A* and *NaPi‐lib* was observed in PGR piglets compared with the healthy piglets (*P* < .05). In the ileal mucosa, mRNA levels of *SLC1A1*, *SLC7A7*, *SLC7A9*, *PEPT1*, *FABP4*, *SLC5A1*, *GLUT2* and two mineral transporters were lower in PGR piglets than those in healthy piglets (*P* < .05), while no significant change was observed in fatty acids transporters (*P* > .05).

**TABLE 1:** Serum concentrations of free amino acids in piglets^1^

Items, μg/g	Healthy	PGR	SEM	*P*‐value
Asp	10.39	8.23	0.95	.040
Thr	68.01	50.15	4.71	.003
Ser	20.47	13.79	1.92	.004
Glu	77.04	62.31	8.26	.108
Gly	125.79	54.03	8.83	<.01
Ala	59.91	45.39	5.82	.026
Cys	6.93	13.16	2.40	.021
Val	27.64	28.35	3.43	.838
Met	6.35	4.7	0.88	.082
Ile	16.37	12.87	2.13	.123
Leu	26.22	20.37	2.55	.038
Tyr	15.15	6.07	1.13	<.01
Phe	19.54	21.18	1.98	.422
Lys	21.96	26.97	3.98	.228
His	8.74	9.46	1.16	.552
Arg	7.01	1.96	0.67	<.01
Trp	23.04	24.92	2.77	.508
Pro	19.02	10.37	1.39	<.01

Abbreviaion: PGR, postnatal growth retardation.

^1^ SEM, pooled SEM; n = 6.

### 3.2 Intestinal mucosal digestive enzyme concentration in PGR piglets

**FIGURE 2:**
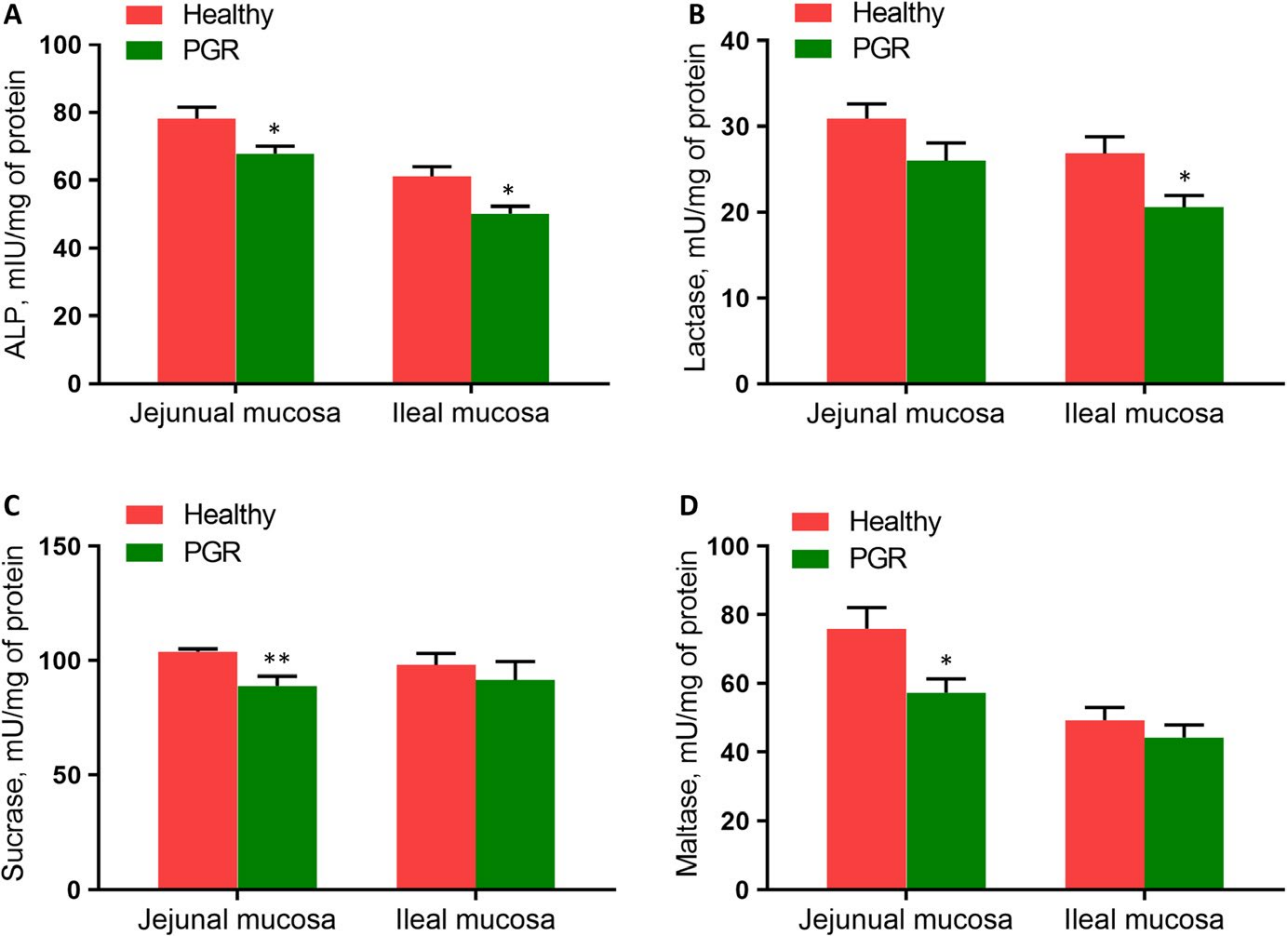
Digestive enzyme activities in the jejunal and ileal mucosa of healthy and PGR piglets. Data are expressed as means ± SEM, n = 6. *, *P* < .05; **, *P* < .01

The results of tested enzyme concentration in the jejunal and ileal mucosa of healthy and PGR piglets were shown in Figure 2. In the jejunal mucosa, the PGR piglets had lower levels of ALP, sucrase and maltase than the healthy group (*P* < .05). In the ileal mucosa, the concentration of ALP and lactase was decreased in the PGR piglets compared with that in the healthy piglets (*P* < .05).

### 3.3 Changed intestinal mitochondria function in PGR piglets

**FIGURE 3:**
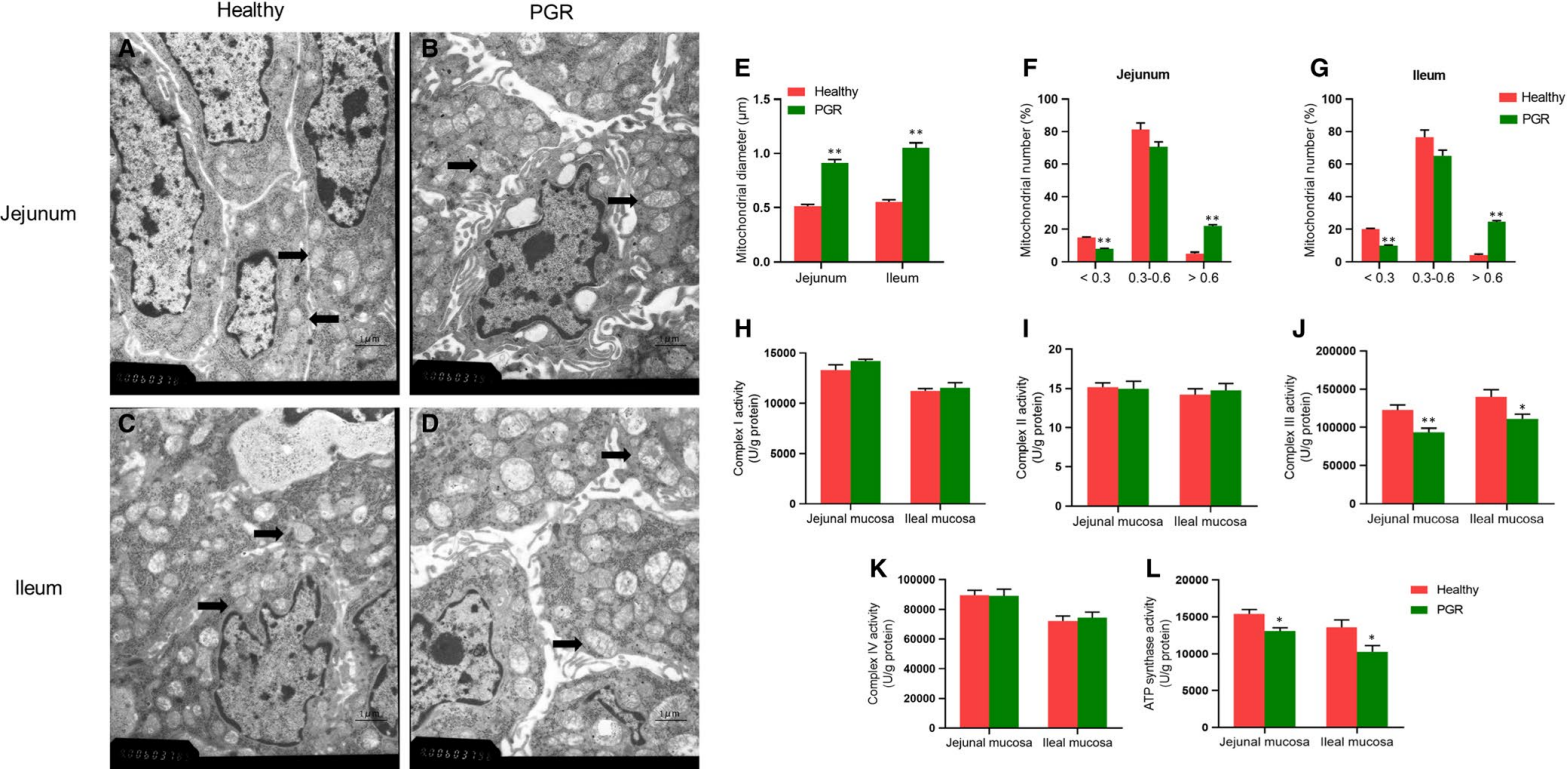
The mitochondria structure (marked with black arrows) and mitochondrial ETC complexes and ATP synthase activities in the jejunal and ileal mucosa of healthy and PGR piglets. A‐D, mitochondria structure in jejunum and ileum; E) mitochondrial diameter; (F, G) distribution of mitochondrial diameter in jejunum and ileum. H‐L, activities of mitochondrial complexes in jejunal and ileal mucosa. Data are expressed as means ± SEM, n = 6. *, *P* < .05; **, *P* < .01

**FIGURE 4:**
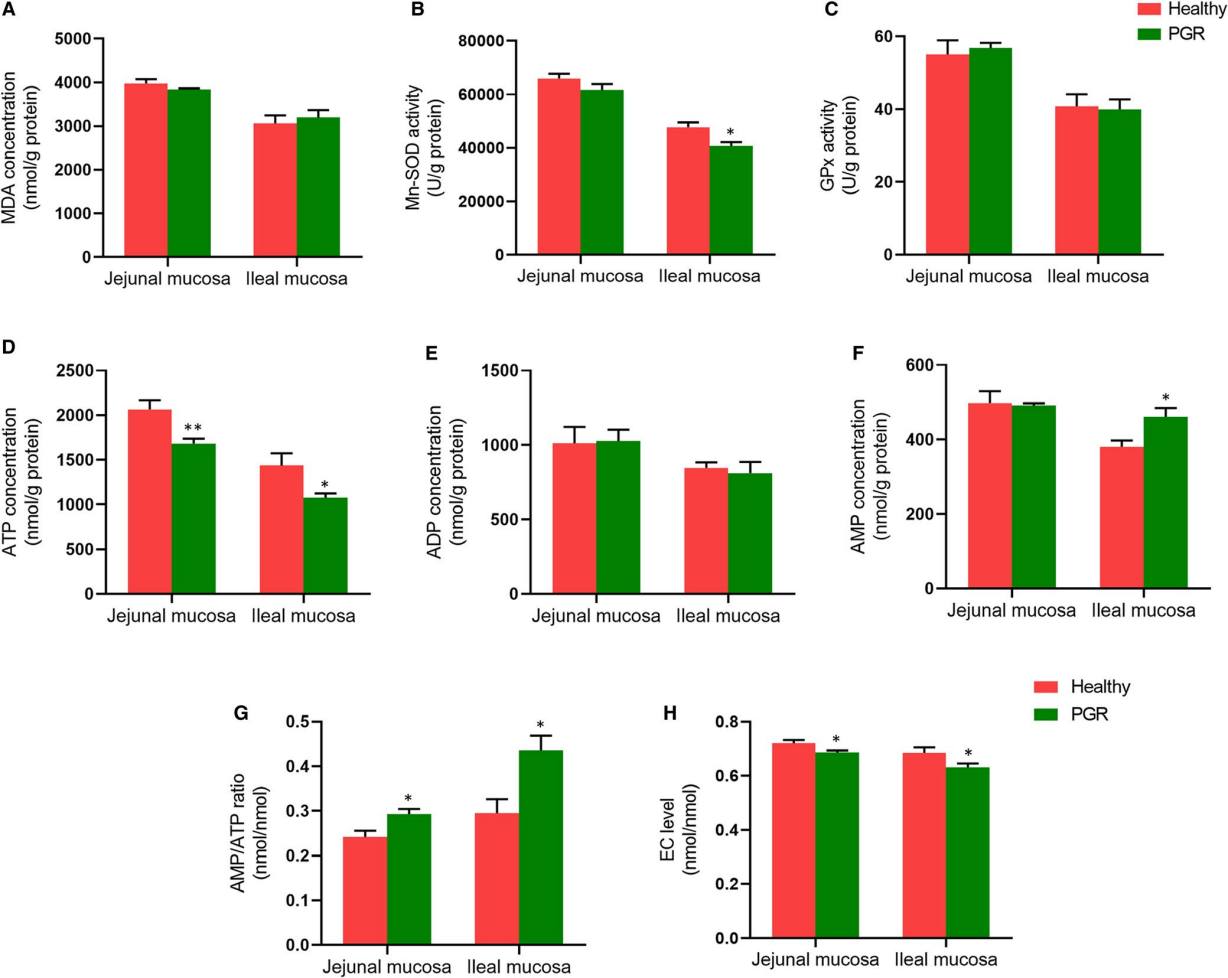
The intestinal mucosal energy status and mitochondrial redox status in the jejunal and ileal mucosa of healthy and PGR piglets. A‐C, mitochondrial redox status in jejunal and ileal mucosa; (D‐H) intestinal mucosal energy status. AMP, adenosine monophosphate; ADP, adenosine diphosphate; ATP, adenosine triphosphate; EC, energy charge. MDA, malondialdehyde; GPx, glutathione peroxidase; SOD, superoxide dismutase. Data are expressed as means ± SEM, n = 6. *, *P* < .05; **, *P* < .01

The mitochondria ultrastructure of the jejunum and ileum in the healthy and PGR piglets was shown in Figure 3A‐D (marked with black arrows). Mitochondria had a normal ultrastructure in the jejunum and ileum of healthy piglet as shown in Figure 3A,C, respectively. However, PGR piglets displayed swollen mitochondria in jejunum and ileum compared with healthy piglets in TEM analysis (*P* < .01) (Figure 3E). Quantitative analysis revealed that more than 20% of mitochondria were swollen in the jejunum and ileum of PGR piglets (diameter >0.6 μm, 5% in healthy piglets, *P* < .01) (Figure 3F,G). The activities of mitochondrial ETC complexes and ATP synthase in two groups were shown in Figure 3H‐L, the activities of mitochondrial ETC complexes III and ATP synthase were significantly decreased in the jejunal and ileal mucosa of PGR piglets compared with healthy piglets (*P* < .05). The mitochondrial redox status in jejunal and ileal mucosa of healthy and PGR piglets was presented in Figure 4A‐C. In jejunal mucosa, no significant difference was observed in MDA level, Mn‐SOD and GPx activity between PGR and healthy piglets (*P* > .05). In the ileal mucosa, PGR piglets exhibited significantly lower activity of Mn‐SOD compared with healthy piglets (*P* < .05).

### 3.4 Changed intestinal mucosal energy status in PGR piglets

**FIGURE 5:**
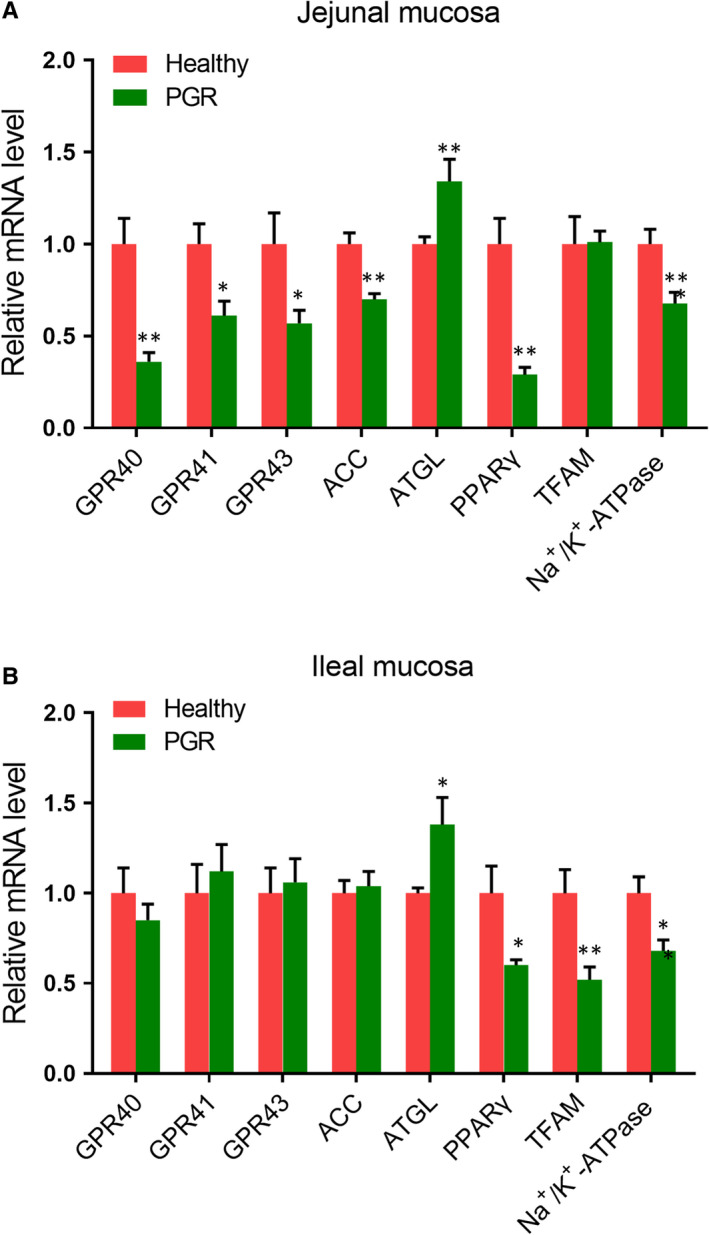
Relative mRNA abundances involved in regulation of energy status in the jejunal (A) and ileal (B) mucosa of healthy and PGR piglets. Data are expressed as means ± SEM, n = 6. *, *P* < .05; **, *P* < .01

**FIGURE 6:**
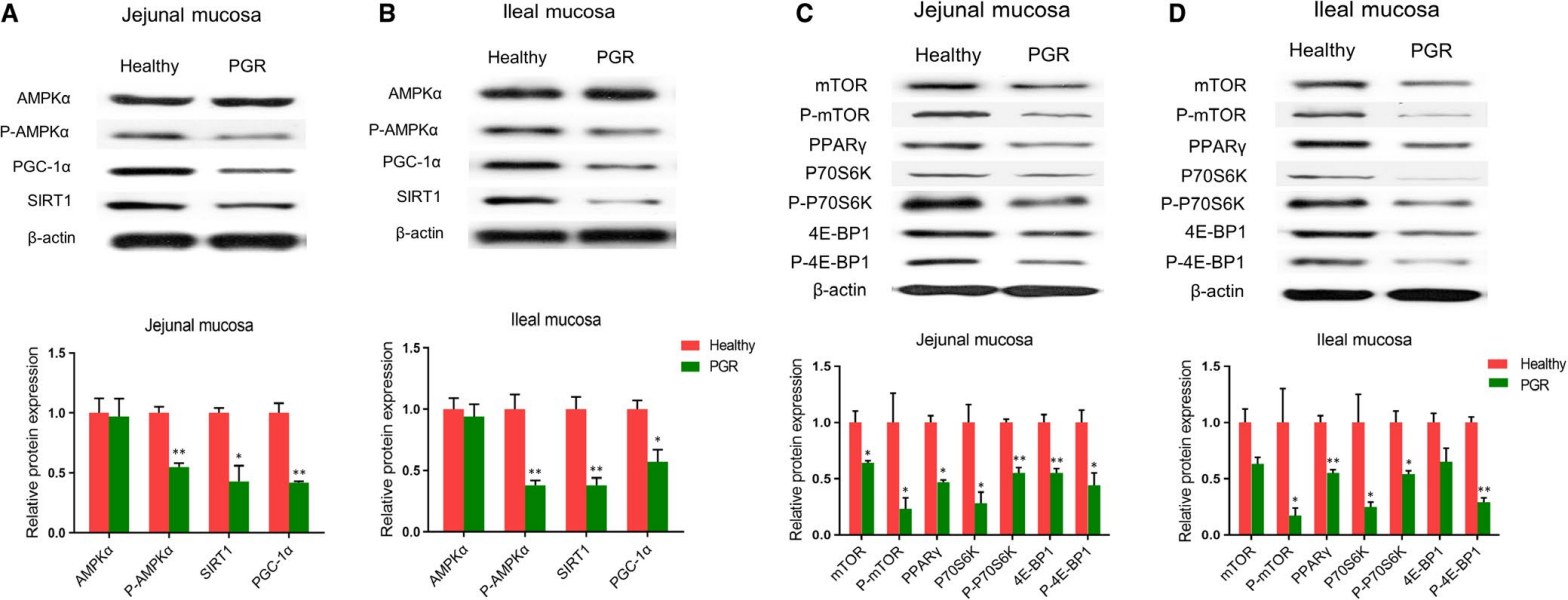
The relative protein abundances involved in AMPK pathway (A, B) and mTOR pathway (C, D) in the jejunal and ileal mucosa of healthy and PGR piglets. Data are expressed as means ± SEM, n = 6. *, *P* < .05; **, *P* < .01

The energy status in the intestinal mucosa of healthy and PGR piglets was shown in Figure 4D‐H. In the jejunal mucosa, markedly lower ATP level (*P* < .01) and EC value (*P* < .05) but higher AMP/ATP ratio (*P* < .05) was observed in PGR piglets as compared with those in healthy piglets. In the ileal mucosa, ATP concentration and EC value were lower, whereas AMP content and AMP/ATP ratio were increased in PGR piglets compared with those in healthy piglets (*P* < .05). Furthermore, we measured mRNA and protein abundances involved in energy metabolism in intestinal mucosa. As shown in Figure 5A‐B, in the jejunal mucosa, compared with healthy piglets, *GPR40*, *GPR41*, *GPR43*, *ACC*, *PPARγ* and *SIRT1* mRNA expressions were decreased; but the mRNA abundances of *AGTL* were increased in PGR piglets (*P* < .05). In the ileal mucosa, the mRNA expression of *PPARγ*, *TFAM* and *Na^+^/K^+^‐ATPase* was decreased; but *ATGL* was increased in PGR piglets (*P* < .05). Additionally, protein expression involved in AMPK and mTOR pathway was also evaluated between healthy and PGR piglets. As shown in Figure 6, in the jejunal mucosa, P‐mTOR, mTOR, PPARγ, P‐4E‐BP1, 4E‐BP1, P‐P70S6K, P70S6K P‐AMPKα, PGC‐1α (*P* < .01) and SIRT1 (*P* < .05) abundances were significantly lower in PGR piglets than those in healthy piglets; while no significant difference was observed in AMPKα expression between two groups (*P* > .05). In the ileal mucosa, compared with healthy piglets, protein expression of P‐AMPK, SIRT1 (*P* < .01), PGC‐1α, P‐mTOR, PPARγ, P‐4E‐BP1, P‐P70S6K, P70S6K (*P* < .05) was down‐regulated in PGR piglets.

## 4 DISCUSSION

**FIGURE 7:**
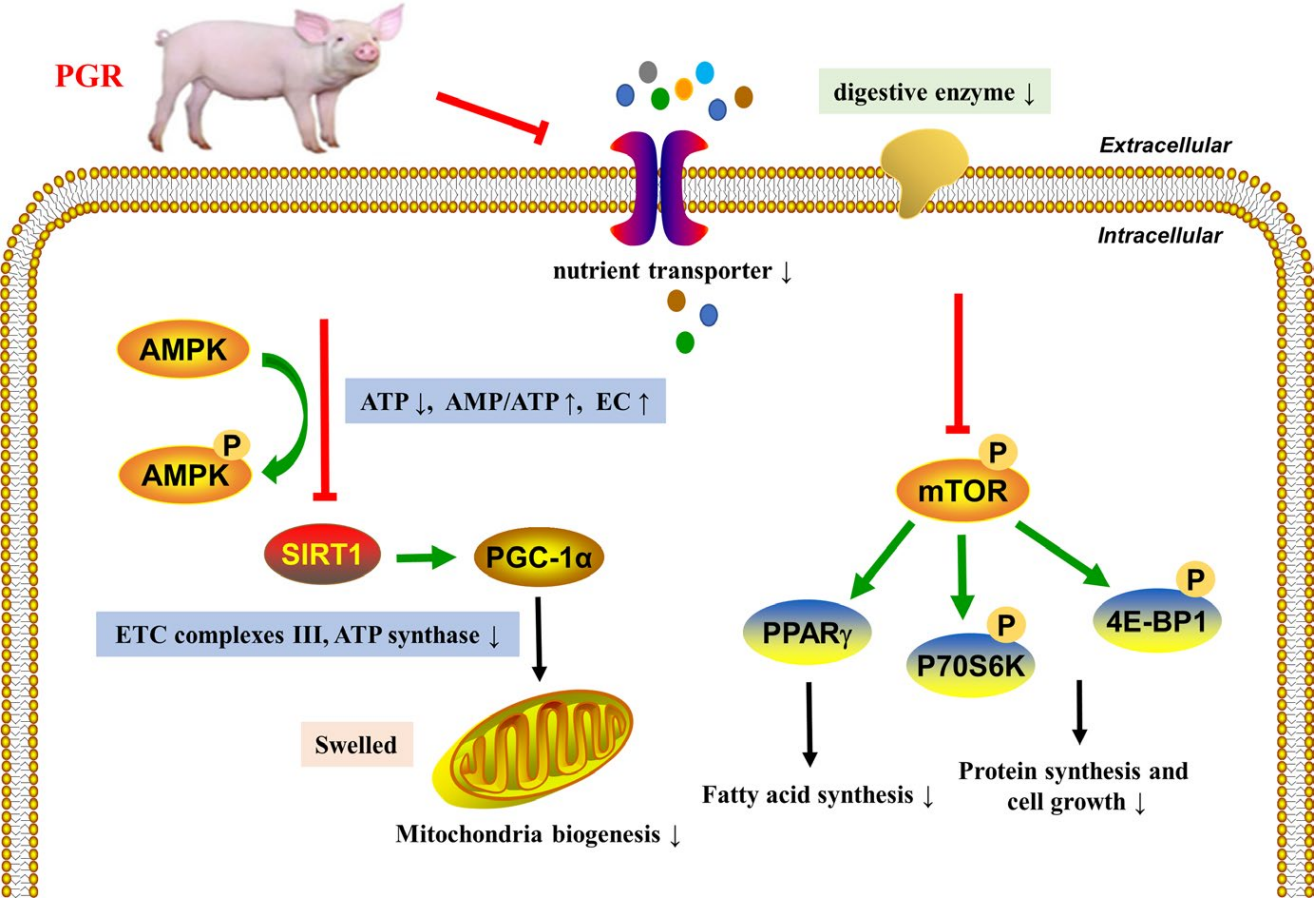
Legend of graphical abstract showing the main results obtained in the current work. The green arrows (↑) indicate direct stimulatory modification, and the red symbols (⊥) indicate direct inhibitory modification. ETC, electron transfer chain; EC, energy charge

PGR piglets are predisposed to malfunction and suffer from mal‐development of the small intestine.^30^ Normal intestinal function is required for maintenance of absorption and transport of nutrients, energy metabolism and protein synthesis.^31^ In the present study, the presence of mitochondrial dysfunction, reduced concentration of digestive enzymes, down‐regulated gene expression involved in nutrient transporters, and aberrant protein expression associated with energy metabolism was observed in the intestinal mucosa of PGR piglets (Figure 7). This is the first study to our knowledge in analysing the difference of intestinal mucosal nutrient absorption and energy metabolism between the healthy and PGR piglets.

We firstly measured serum biochemical indexes. TG, CHOL, HDL and LDL concentrations were significantly decreased in PGR piglets, suggesting that PGR may impair lipid metabolism. It could be evidenced by the reduced expression levels of some tested lipid transporters and receptors in the intestinal mucosa of PGR piglets. Meanwhile, low levels of serum calcium and phosphorus, which is in accordance with the decreased mRNA expressions of calcium and phosphorus (NaPi‐lib and Calbindin D9k) transporters were observed in the jejunal and ileal mucosa of PGR piglets. It was agreed with the lower serum concentration of calcium and phosphorus in infants with IUGR.^32^

An increase in the amount of amino acids in portal vein is as key regulators of metabolic pathways in postnatal development.^33^ In this study, the contents of most determined amino acids we tested were decreased in PGR piglets compared with healthy piglets, of which essential amino acids represented the most. Those changed amino acids were served as a series of physical, metabolic and nutritional functions including energy supplementation, modulation of immune function, hormone secretion, cytokine production and anti‐oxidative function.^33^, ^34^, ^35^ The decreased serum levels of amino acids may be because of the reduced food intake and dysfunction of intestinal absorption. It coincided with that most of amino acids transporters in the jejunal and ileal mucosa were down‐regulated in PGR piglets, which indicated that down‐regulated expression of amino acids transporters in the intestinal mucosa led to the disrupted serum amino acids metabolism in PGR piglets.

In addition, we measured digestive enzyme concentration in the intestinal mucosa of PGR piglets. Intestinal ALP serves as a key marker enzyme when considering changes in intestinal digestive and absorptive functions.^36^ Our results showed reduced level of ALP in the jejunal and ileal mucosa of PGR piglets. In addition, some stress such as early weaning and IUGR have been reported to decrease the activities of jejunal and ileal lactose, sucrose and maltase.^37^, ^38^ In our study, reduced concentration of sucrose and maltase in jejunal mucosa, and lactase in ileal mucosa were observed in PGR piglets as compared with healthy piglets. It suggested that impaired intestinal digestive and absorptive functions may cause PGR.

Next, we explored the gene expression involved in nutrient transporters in intestinal mucosa of PGR piglets. FATPs and FABPs are families of proteins which play key roles in fatty acid uptake and activation.^39^, ^40^ Glucose uptake in the intestinal epithelial cells depended on two types of glucose transporters: the apically expressed SLC5A1 and the basolaterally expressed GLUT2.^41^ We found decreased mRNA abundance of FATP1 and FABP4 in jejunal mucosa, and FABP4, SLC5A1 and GLUT2 in ileal mucosa of PGR piglets. Amino acids and peptide transporters showed the same trend in PGR piglets. These results coincided with previous study that PGR mice exhibited decreased levels of glucose and fatty acids transporters in the small intestine.^42^, ^43^ Our resulted suggested that declined intestinal absorptive capacity may contribute to PGR piglets.

Further, mitochondrial damage, particularly ETC complexes, contributed to growth retardation in clinical and experimental model.^19^, ^44^ In our study, we did TEM study for mitochondria structure, and the swelled mitochondria were observed in jejunum and ileum of PGR piglets. The occurrence of swelled mitochondria may suggest a weakened antioxidant capacity and abnormal ability of ATP production or transport, which may be partly responsible for damage to the small intestine in PGR piglets.^45^ In addition, the present study showed the lower activity of ETC complex III and ATP synthase in the intestinal mucosa of PGR piglets, which was in accordance with previous study that ATP production and activities of complex IV were significantly decreased in cord blood mononuclear cells of IUGR patients.^46^ Complex III system is associated with generating superoxide radicals, indicating that PGR piglets may display impaired mitochondrial antioxidant system, which was supported by the finding that the activity of Mn‐SOD was decreased in the ileal mucosa of PGR piglets. It agreed with previous study that IUGR induced accumulation of reactive oxygen species in liver of piglet.^21^ The activity of Mn‐SOD was lower in the jejunum of IUGR piglets.^47^ Besides, we observed decreased ATP production and increased AMP/ATP ratio in intestinal mucosa of PGR piglets as compared with healthy piglets. The observed effect was consistent with the decreased activity of ATP synthase in intestinal mucosal mitochondria. Meanwhile, the abnormal expression of TFAM, a key regulator of mitochondrial transcription, was also observed in ileal mucosa of PGR piglets. It suggested that intestinal mucosal mitochondrial dysfunction may be associated with PGR piglets.

Generally, AMPKα served as an energy sensor in response to alterations in cellular energy status, which was activated by the elevations in AMP/ATP ratio.^48^ However, in the PGR piglets, the intestinal mucosal AMPK was not activated by the increased ratio of AMP/ATP. Instead, the down‐regulated phosphorylation level of AMPKα was observed in PGR piglets, which was in accordance with the lower hepatic protein abundance of P‐AMPKα in IUGR piglets.^21^ Although the exact mechanism underlying this observation had not been determined, decreases in the activity of SIRT1 and PGC‐1α may be implicated in this process. SIRT1, served as a key mediator of mitochondrial biogenesis, has been involved in the regulation of energy metabolism via deacetylation of PGC‐1α.^49^ Increasing SIRT1 expression could modulate PGC‐1α expression in vivo and finally regulated energy homeostasis.^50^ In our study, the protein expression of SIRT1 and PGC‐1α was significantly decreased in intestinal mucosa of PGR piglets, which agreed with the previous study that IUGR rats induced decreased transcriptional levels of SIRT1 and PGC‐1α in the skeletal muscle.^51^ In addition, IUGR piglets also displayed reduced hepatic gene expression associated with mitochondrial biogenesis compared with normal‐birth‐weight piglets.^20^ These results indicated that deteriorated mitochondrial biogenesis function may lead to PGR piglets, and the SIRT1/PGC‐1α axis may become a potential therapeutic target for intestinal mucosal disorder energy states.

Furthermore, mTOR played a key role in regulating many fundamental cell processes, including protein synthesis and cell proliferation. Suppression of mTOR signalling may inhibit fatty acids and protein synthesis or promote lipolysis, resulting in the decrease of total fat weight. mTOR promoted protein synthesis largely through activating the expression of 4E‐BP1 and P70S6K.^52^ In our study, the down‐regulated expression of P‐mTOR, P‐ P70S6K and P‐4E‐BP1 was observed in intestinal mucosa of PGR piglets. These results agreed with the previous studies that IUGR piglets experienced the reduced protein synthesis in the foetal gut.^31^ The PPARγ and ATGL played important roles in process of fatty acids synthesis and lipolysis. A decrease in protein abundance of PPARγ, accompanied by the increased ATGL mRNA expression, was observed in intestinal mucosa of PGR piglets, which suggesting PGR piglets with lower capacity of fat deposition. Activation of placental PPARγ protected against PGR mice in offspring.^53^ In addition, the activation of Na^+^/K^+^‐ATPase helped coordinate food digestion and nutrient absorption, correct the impaired mitochondrial energy status, and regulate energy metabolism and immune response.^54^, ^55^, ^56^ In the present study, significantly decreased mRNA abundance of Na^+^/K^+^‐ATPase was observed in the jejunal and ileal mucosa of PGR piglets, which suggested that PGR piglets may display disrupted energy metabolism via inhibition of nutrients absorption and mitochondrial function. Moreover, abnormal mRNA expression of GPR40, GPR41 and GPR43 led to metabolic disorders. They were reported to be involved in energy regulation in response to short‐chain fatty acids (SCFAs) generated by gut microbiome.^57^, ^58^ Our results showed reduced mRNA expression of GPR40, GPR41 and GPR43 in the jejunal mucosa of PGR piglets, which was in accordance with the disordered energy metabolism of PGR piglets. However, the interaction between the host and the gut microbiome needs to be further studied. In line with these findings, our data suggested that PGR piglets experienced impaired ability of protein synthesis and lipid metabolism via the down‐regulation of mTOR pathway in the intestinal mucosa.

In summary, the current study provided the novel evidence for the alteration of the nutrient absorption and energy metabolism in the intestinal mucosa of PGR piglets. Specifically, our findings revealed the decreased digestive enzyme activities, low capacity for mitochondrial biogenesis and ETC system, altered gene expression of nutrient transporters, and deteriorated energy homeostasis in the intestinal mucosa of PGR piglets. The aberrant energy status was likely associated with compromised AMPKα/mTOR pathway. Although the sampling time point was limited to d 42 after birth, the findings still provide valuable information to better understand the mechanisms of disorder of energy status in PGR piglets and provide new ideas for the nutritional intervention strategies.

## CONFLICT OF INTEREST

The authors declare that they have no conflict of interest.

## AUTHOR CONTRIBUTION

**Ming Qi:** Conceptualization (equal); Data curation (lead); Formal analysis (lead); Writing‐original draft (lead). **Jing Wang:** Conceptualization (equal); Funding acquisition (equal); Writing‐review & editing (equal). **Bie Tan:** Conceptualization (equal); Funding acquisition (equal); Project administration (equal); Writing‐review & editing (equal). **Simeng Liao:** Methodology (equal); Visualization (equal). **Cimin Long:** Methodology (equal); Visualization (equal). **Yulong Yin:** Conceptualization (equal); Funding acquisition (equal); Writing‐review & editing (equal).

## Supporting information

Table S1

## Data Availability

All data generated or analysed during this study are included in this published article and its supplementary information files.
