# Supplementary material for: Postnatal growth retardation is associated with intestinal mucosa mitochondrial dysfunction and aberrant energy status in piglets
Source: J Cell Mol Med. 2020 Jul 15;24(17):10100–11. doi: 10.1111/jcmm.15621 (PMC7520312; doi:10.1111/jcmm.15621)
Supplement: Supplementary file 1 — Table S1 [file JCMM-24-10100-s001.docx]

| Gene | Gene Bank NO. | Sequence | Product length, bp |
| --- | --- | --- | --- |
| *β-actin* | XM_0031242803 | F:CTGCGGCATCCACGAAACT | 147 |
|  |  | R:AGGGCCGTGATCTCCTTCTG |  |
| *SLC7A7* | NM_001110421.1 | F:TTTGTTATGCGGAACTGG | 155 |
|  |  | R:AAAGGTGATGGCAATGAC |  |
| *SLC7A9* | NM_001110171.1 | F:GAACCCAAGACCACAAATC | 180 |
|  |  | R:ACCCAGTGTCGCAAGAAT |  |
| *SLC7A1* | NM_001012613.1 | F:TCTGGTCCTGGGCTTCATAA | 123 |
|  |  | R:ACCTTCGTGGCATTGTTCAG |  |
| *SLC38A2* | NM_001317081.1 | F:GTTACCTTTGGTGATCCAGGC | 96 |
|  |  | R:ACCAATGACACCAGCAGAACC |  |
| *PepT1* | NM_214347.1 | F:CAGACTTCGACCACAACGGA | 99 |
|  |  | R:TTATCCCGCCAGTACCCAGA |  |
| *ASCT2* | XM_003127238.5 | F:CTGGTCTCCTGGATCATGTGG | 207 |
|  |  | R:CAGGAAGCGGTAGGGGTTTT |  |
| *SLC1A1* | NM_001164649.1 | F:GGCACCGCACTCTACGAAGCA | 177 |
|  |  | R:GCCCACGGCACTTAGCACGA |  |
| *GPR41* | NM_001315601.1 | F:TGGAGACCTTACGTGTTG | 75 |
|  |  | R:CGAGGATGAGAAGTAGTAGAT |  |
| *GPR43* | NM_001278758.1 | F:CGTGTTCATCGTTCAGTA | 76 |
|  |  | R:GAAGTTCTCATAGCAGGTA |  |
| *GPR40* | NM_001278783.1 | F:TGCTCTGACCTCCTGCTGG | 235 |
|  |  | R:CACACACCCCCCAGGAATAG |  |
| *SGLT1* | NM_001164021.1 | F:TCATCATCGTCCTGGTCGTCTC | 144 |
|  |  | R:CTTCTGGGGCTTCTTGAATGTC |  |
| *GPRC6A* | XM_021089187.1 | F:CTTGAGAAAATCATAGCAGAAGCC | 161 |
|  |  | R:GGAATGGTAGTTATCTTGGTGGC |  |
| *GLUT2* | NM_001097417.1 | F:ATTGTCACAGGCATTCTTGTTAGTCA | 273 |
|  |  | R:TTCACTTGATGCTTCTTCCCTTTC |  |
| *NaPi-Iib* | NM_001256772.1 | F:CTCTGTAGCTGCCGGGTCCTAA | 198 |
|  |  | R:GGTCAGAGTCGACGAGAACAC |  |
| *Calbindin D9k* | NM_001164021.1 | F:AGAGCAAATGCACCTCTTGG | 105 |
|  |  | R:CATGTGAGCGCATAGAAGGA |  |
| *GPR40* | NM_001278783.1 | F:TGCTCTGACCTCCTGCTGG | 235 |
|  |  | R:CACACACCCCCCAGGAATAG |  |
| *GPR41* | NM_001315601.1 | F:TGGAGACCTTACGTGTTG | 75 |
|  |  | R:CGAGGATGAGAAGTAGTAGAT |  |
| *GPR43* | NM_001278758.1 | F:CGTGTTCATCGTTCAGTA | 76 |
|  |  | R:GAAGTTCTCATAGCAGGTA |  |
| *ACC* | NM_001114269.1 | F:ATGTTTCGGCAGTCCCTGAT | 133 |
|  |  | R:TGTGGACCAGCTGACCTTGA |  |
| *ATGL* | NM_001098605.1 | F:GCCCACGAGTGATAGCATCC | 109 |
|  |  | R:CAGCAGGTTGGACAGGGTG |  |
| *PPARγ* | NM_214379.1 | F:ATTTACACCATGCTGGCCTC | 123 |
|  |  | R:GGGCTCCATAAAGTCACCAA |  |
| *TFAM* | NM_001130211.1 | F:CCACCCTGAGTGGTTTTCCA | 193 |
|  |  | R:TGCCAGTCTGCCCTATAAGC |  |
| *Na+/K+-ATPase* | NM_214249.1 | F:TCGATAATCTCTGCTTCGTTGG | 142 |
|  |  | R:ATGGCTTTGGCTGTGATGG |  |
